# Kombucha-Derived Bacterial Cellulose for Active and Biodegradable Food Packaging: Production, Modification, Performance, and Current Challenges

**DOI:** 10.3390/molecules31173131

**Published:** 2026-09-07

**Authors:** Joanna Maria Jasińska, Ewelina Jamróz

**Affiliations:** 1 Department of Chemistry, University of Agriculture, Balicka Street 122, 30-149 Krakow, Poland; 2 Department of Product Packaging, Krakow University of Economics, ul. Rakowicka 27, 31-510 Krakow, Poland

**Keywords:** kombucha, bacterial cellulose, SCOBY, food packaging, films, coatings

## Abstract

Kombucha fermentation generates two fractions of potential relevance to food-packaging applications: a fermented liquid containing organic acids, polyphenols and other metabolites, and a cellulose-rich pellicle formed by cellulose-producing acetic acid bacteria. However, the fermented beverage, raw pellicle and purified kombucha-derived bacterial cellulose (KBC) differ substantially in composition, functionality and food-contact suitability and should not be treated as interchangeable materials. This review critically examines bacterial cellulose formation during kombucha fermentation, purification and modification strategies, packaging-relevant properties, and the application of kombucha-derived materials in films, coatings and composites. Purified KBC provides a continuous nanofibrillar network and can exhibit good mechanical and oxygen-barrier properties under dry conditions. Its hydrophilic character, however, promotes moisture sorption and swelling, which may impair mechanical integrity and barrier performance at elevated relative humidity. Blending, coating, grafting and crosslinking can improve selected material properties, but their effects are formulation-specific and may involve trade-offs between moisture resistance, mechanical performance, biodegradability and food-contact safety. Fermented kombucha liquid and native or mildly washed pellicles may contribute fermentation-derived bioactive compounds; however, antioxidant or antimicrobial activity measured in the beverage or raw pellicle cannot be automatically attributed to purified KBC or the final packaging material. Current evidence supports the use of KBC as a structural matrix, reinforcing phase, coating component or carrier of active substances. Nevertheless, limited migration data, heterogeneous testing conditions, insufficient real-food studies and a lack of pilot-scale, regulatory and life-cycle assessments currently restrict its broader industrial implementation.

## 1. Introduction

Kombucha is a fermented beverage traditionally produced by inoculating a sweetened tea infusion, most commonly prepared from black or green tea (*Camellia sinensis* L.), with a symbiotic culture of bacteria and yeasts, commonly referred to as SCOBY [1,2,3,4,5,6]. During fermentation, the microorganisms convert sugars into ethanol, carbon dioxide, organic acids, and other metabolites. Simultaneously, cellulose-producing acetic acid bacteria synthesise an extracellular nanofibrillar network that accumulates at the air–liquid interface and forms a characteristic floating pellicle [4,7,8].

Although the pellicle is commonly referred to as SCOBY, a distinction should be made between the microbial consortium, the raw fermentation pellicle, and purified bacterial cellulose [8,9,10,11]. The SCOBY consortium comprises microorganisms involved in fermentation, whereas the raw pellicle contains bacterial cellulose together with microbial cells, fermentation metabolites, and residues originating from the cultivation medium. Purified bacterial cellulose is obtained after the removal of microbial biomass and non-cellulosic components. These materials may therefore differ substantially in composition, functionality, safety, and suitability for food-contact applications [8,9,10,11].

Bacterial cellulose has attracted increasing attention as a renewable material for sustainable food packaging because of its highly organised nanofibrillar structure, mechanical strength, film-forming capacity, high purity after appropriate treatment, and potential biodegradability [11,12,13,14]. However, the abundance of hydroxyl groups in the cellulose structure results in pronounced hydrophilicity, water absorption, and swelling. Exposure to humid conditions may consequently reduce the mechanical strength and water-vapour and oxygen-barrier properties of bacterial cellulose films [12,14]. Various modification strategies have therefore been investigated, including blending bacterial cellulose with poly(vinyl alcohol), chitosan, and other polymers, hydrophobic surface modification and esterification, chemical crosslinking, and the development of films exhibiting antimicrobial or antioxidant activity [11,12,13,14].

The fermented kombucha liquid contains organic acids, polyphenols, and other fermentation-derived compounds whose concentrations depend on the substrate, microbial composition, temperature, and fermentation time [15,16,17,18]. In addition, some of these compounds, together with microbial cells and medium-derived residues, may remain within the untreated kombucha pellicle [10,19]. Native films produced directly using kombucha microbial cultures and different plant infusions have been reported to exhibit measurable biological activity [20]. Nevertheless, antioxidant or antimicrobial activity demonstrated in the fermented beverage cannot be directly attributed to the raw pellicle, purified bacterial cellulose, or final packaging material. The retention, stability, migration, and functional effectiveness of these compounds should therefore be determined directly at each stage of material preparation.

Previous reviews on kombucha have primarily addressed beverage fermentation, chemical composition, microbial ecology, and potential health-related effects [21,22,23]. Other reviews have considered bacterial cellulose as a general material for food and food-packaging applications, without focusing specifically on bacterial cellulose produced by the kombucha consortium and the consequences of its purification and processing [23,24]. Comparatively less attention has therefore been devoted to the relationship between kombucha-derived bacterial cellulose production, purification, modification, and measurable food-packaging performance. Furthermore, published studies differ considerably in terms of microbial cultures, fermentation substrates, purification procedures, drying and conditioning conditions, and analytical methods, which limits direct comparison of the reported results [10,11,13,14,20].

This review evaluates the potential of kombucha-derived bacterial cellulose for active and biodegradable food-packaging applications. Particular attention is given to bacterial cellulose formation during kombucha fermentation, differences between raw SCOBY pellicles and purified bacterial cellulose, purification and modification strategies, packaging-relevant properties, and applications tested using model systems or real food products. The principal technological, economic, regulatory, and scale-up limitations are also discussed to identify realistic future applications and priorities for further research.

## 2. Fermentation and Formation of Bacterial Cellulose

**Figure 1:**
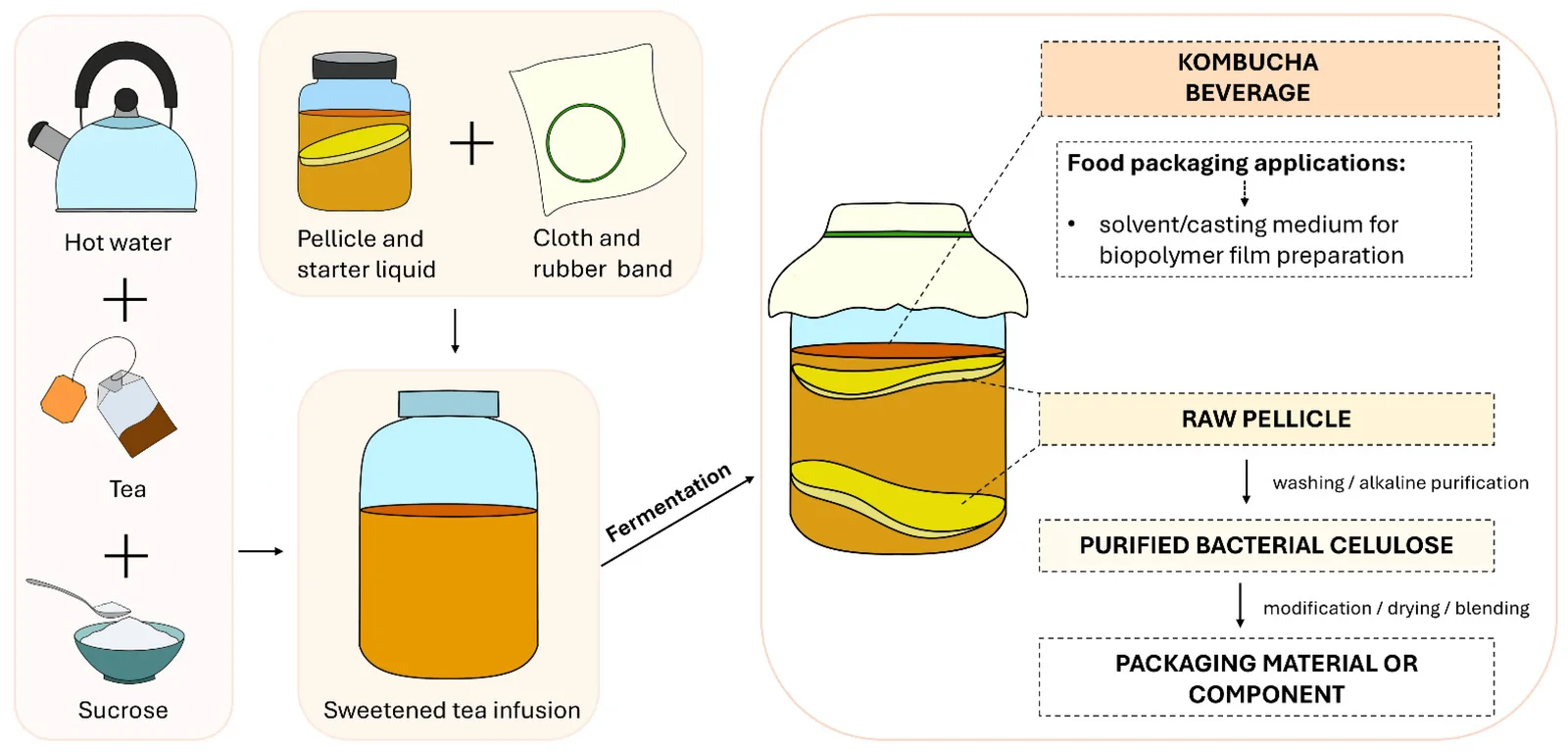
Schematic representation of kombucha preparation and the distinct processing routes of fermented kombucha liquid and the raw cellulose pellicle for food-packaging applications.

Kombucha fermentation is carried out by a complex symbiotic consortium of bacteria and yeasts. Its composition varies considerably, depending on the geographical origin and history of the inoculum, the type of substrate, cultivation conditions, and analytical method used for microbial identification [3,22,25]. The bacterial fraction generally includes acetic acid bacteria, particularly species belonging to the genera *Komagataeibacter*, *Gluconobacter*, and *Acetobacter*. Lactic acid bacteria, including species of *Lactobacillus* and *Lactococcus*, may also be present, although their abundance is highly variable. The yeast community may comprise species belonging to *Saccharomyces*, *Zygosaccharomyces*, *Brettanomyces*, *Schizosaccharomyces*, *Torulaspora*, *Saccharomycodes*, and other genera [22,25,26,27]. A schematic representation of kombucha preparation is presented in Figure 1.

The starter culture may consist of fermented kombucha liquid, a portion of the cellulose pellicle, or a combination of both. Microorganisms are present both in the liquid phase and within the floating cellulose-based matrix [8,9]. However, the distribution of microbial populations between these phases may differ. Yeasts and bacteria suspended in the liquid participate directly in substrate conversion, whereas cells embedded in the pellicle form a surface-associated microbial community with access to atmospheric oxygen. Traditional kombucha is produced from sweetened black or green tea; however, coffee, herbal infusions, fruit juices, seaweed extracts, and food-industry by-products have also been investigated as alternative fermentation substrates [28,29,30,31,32,33]. The composition of the cultivation medium can influence microbial growth, metabolite production, cellulose yield, cellulose morphology, and the final properties of the obtained material. Alternative substrates may reduce production costs and support circular-economy approaches; however, they may also increase batch-to-batch variability and introduce additional compounds that require removal during purification.

### 2.1. Metabolic Interactions During Fermentation

Kombucha fermentation involves several interconnected metabolic pathways, primarily alcoholic and acetic fermentation, with lactic fermentation occurring in cultures containing sufficient populations of lactic acid bacteria [22]. The process is initiated by yeasts, which hydrolyse sucrose into glucose and fructose using invertase enzymes. These monosaccharides are subsequently metabolised to produce ethanol and carbon dioxide [34]. Fructose may be preferentially utilised by some yeasts; however, substrate preferences depend on the microbial composition of the culture. Under conditions of high osmotic pressure, yeasts may also produce glycerol. Acetic acid bacteria can subsequently oxidise glycerol to dihydroxyacetone [35]. Yeast metabolism additionally generates volatile compounds and esters that contribute to the aroma of the beverage; however, these sensory aspects are of secondary importance when kombucha fermentation is considered primarily as a method of producing bacterial cellulose [36]. The ethanol generated by yeasts is oxidised by acetic acid bacteria to acetaldehyde and subsequently to acetic acid. Glucose can also be oxidised to gluconic acid and, through further metabolic pathways, to other organic acids, including glucuronic acid [22,35]. These reactions decrease the pH of the fermentation medium and create conditions that inhibit many contaminating and pathogenic microorganisms. Yeast autolysis may additionally release vitamins, amino acids, and other nutrients that support bacterial growth, illustrating the metabolic interdependence between yeasts and bacteria within the consortium [22]. The concentration of fermentation products depends on the tea or alternative substrate, sugar concentration, microbial composition, temperature, oxygen availability, and fermentation time [16,18,37]. Therefore, values reported for organic acids and other metabolites may vary considerably between studies. Direct comparison requires particular caution because concentrations may be expressed using different units and may refer to different fermentation stages.

### 2.2. Formation of the Bacterial Cellulose Pellicle

Bacterial cellulose in kombucha is mainly produced by acetic acid bacteria, especially *Komagataeibacter*, which convert sugars into UDP-glucose, the direct precursor of cellulose. Cellulose synthase then polymerises UDP-glucose into β-1,4-glucan chains that are secreted outside the cell and assemble into a nanofibrillar network [7,38,39]. Under static conditions, cellulose forms a floating pellicle at the air–liquid interface, where oxygen is readily available and supports bacterial metabolism. Cells are mainly located in the upper, oxygen-rich layer, where cellulose is actively produced [39,40,41]. The pellicle may also help maintain cells at this interface and provide some protection, although this role is not fully confirmed in kombucha systems [7,39]. Pellicle formation is gradual; in early stages, cellulose fibrils develop around microbial aggregates, including yeasts, which can act as structural support. Over time, the network thickens into a multilayered structure [7]. Its final properties depend on strain composition, carbon source, oxygen availability, nutrients, and culture conditions. Oxygen diffusion decreases as thickness increases, making cellulose production spatially uneven, with higher activity near the surface [7,39,40,42,43]. Pellicle formation is not driven only by reduced ethanol levels. Ethanol is oxidised by bacteria and can also serve as an additional energy source, indirectly supporting cellulose synthesis from glucose [7,42]. Overall, cellulose production results from combined metabolic interactions, oxygen supply, and cultivation conditions rather than a single factor.

### 2.3. Effect of Process Parameters

Temperature is one of the main factors affecting kombucha fermentation and bacterial cellulose formation. Fermentation generally proceeds at temperatures above approximately 20 °C, while temperatures between 22 and 30 °C are commonly reported as favourable for microbial activity [3,44,45]. Neffe-Skocińska et al. [46] identified 25 °C and a fermentation period of ten days as suitable conditions for obtaining a microbiologically stable beverage with acceptable sensory properties. Nevertheless, conditions optimised for beverage production may not necessarily provide the highest bacterial cellulose yield or the most desirable material properties. Higher temperatures may accelerate sugar utilisation and organic-acid formation, but excessive temperatures can disturb the balance between microbial populations and lead to excessive production of ethanol or acetic acid [47,48]. Temperature may also affect cellulose synthesis, crystallinity, fibre organisation, water retention, and mechanical behaviour. Consequently, studies focused on packaging materials should report not only the fermentation temperature but also the cellulose yield and physicochemical characteristics of the resulting pellicle. The pH decreases during fermentation as organic acids accumulate. Kombucha beverages are commonly reported to reach pH values within the range of approximately 2.5–4.2 [49,50,51,52,53,54]. A decline below pH 4.6 contributes to microbiological safety by limiting the growth of many pathogenic bacteria [55]. However, excessive acidification may alter microbial activity and affect cellulose production. Initial pH, final pH, and changes during fermentation should therefore be controlled and reported in studies concerning bacterial cellulose.

Fermentation time also affects both metabolite accumulation and pellicle thickness. Traditional fermentation usually lasts approximately 7–21 days [56,57], although the optimum duration depends on the desired product. Prolonged fermentation may increase cellulose mass, but after depletion of nutrients or excessive acidification, further cultivation may provide limited benefits. Moreover, longer processing increases production time and costs, which represents an important limitation for industrial scale-up. Oxygen availability is particularly critical because acetic acid bacteria are obligate aerobes [41,43]. In static cultures, oxygen transfer is limited by the surface area of the liquid and the geometry of the vessel. A large surface-to-volume ratio generally supports pellicle formation, whereas deeper fermentation vessels may limit oxygen access and reduce volumetric productivity [41,43]. Although agitation or aeration can improve oxygen transfer, dynamic cultivation may lead to the formation of irregular cellulose particles or aggregates rather than a continuous pellicle. Reactor design must therefore be adapted to the intended form of the final material [43,58]. Other relevant variables include sugar concentration, type of carbon and nitrogen sources, inoculum concentration, age of the starter culture, substrate composition, and the presence of competing microorganisms. Differences in these parameters contribute to substantial variation in cellulose yield and properties among published studies and hinder the development of standardised production protocols [59,60,61].

### 2.4. Primary and Secondary Fermentation

Commercial kombucha production may include two fermentation stages. During primary fermentation, commonly referred to as F1, sweetened tea is fermented in contact with air, resulting in organic-acid production and cellulose-pellicle formation. A secondary fermentation, or F2, may subsequently be conducted in sealed containers with added sugars, fruits, or flavouring ingredients to promote carbonation and flavour development [2,36,62].

From the perspective of bacterial cellulose production, the primary aerobic fermentation is the most relevant stage. Secondary fermentation is primarily used to modify the sensory characteristics of the beverage and generally does not play a central role in the formation of a continuous cellulose pellicle because oxygen availability is restricted. Therefore, detailed discussion of secondary fermentation is not essential when the primary objective is to assess kombucha-derived bacterial cellulose as a packaging material.

### 2.5. From Raw Pellicle to Packaging Material

The freshly harvested kombucha pellicle is not equivalent to purified bacterial cellulose. It consists of a hydrated cellulose network containing bacterial and yeast cells, proteins, polyphenols, and other residues derived from the fermentation medium [11,19]. Its exact composition depends on the substrate and fermentation conditions [11].

For material applications, the pellicle is commonly washed and treated with alkaline solutions, particularly sodium hydroxide, to remove microbial biomass, residual sugars, and other non-cellulosic components [11,19,63]. Repeated washing is subsequently required to remove treatment residues and restore a neutral pH [63]. The purified material may then be dried and further modified, for example, by blending it with other polymers, crosslinking, or incorporating active compounds [10,12,20].

Purification increases the chemical purity of bacterial cellulose but can also remove polyphenols and other fermentation-derived compounds that may contribute to the biological activity of raw SCOBY [19]. Consequently, clear identification of the investigated material—as a raw pellicle, washed pellicle, partially purified membrane or purified bacterial cellulose—is essential for valid comparisons of mechanical, barrier, active and safety-related properties.

## 3. Chemical Composition and Properties of Kombucha

### 3.1. Chemical Composition and Bioactive Compounds of Kombucha and BC Pellicle for Biomaterial Applications

**Figure 2:**
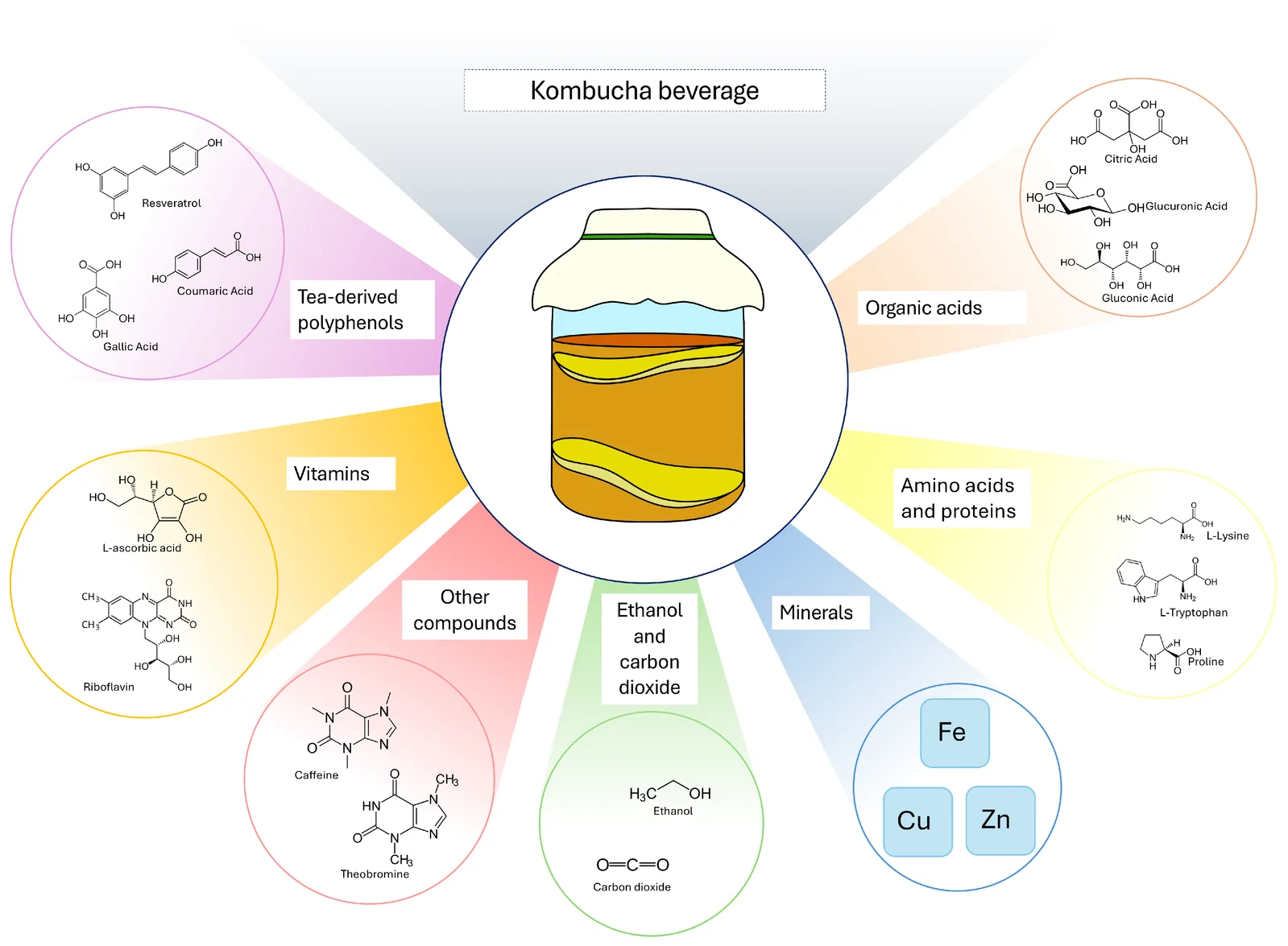
Chemical composition of kombucha beverage.

Chemical analyses have shown that kombucha contains a diverse range of bioactive compounds, including organic acids, sugars, vitamins, amino acids, proteins, minerals, ethanol, carbon dioxide, tea-derived polyphenols, and various metabolites produced during fermentation (Figure 2) [3]. The major organic acids identified in kombucha include acetic, gluconic, glucuronic, citric, lactic, malic, succinic, and pyruvic acids. Its chemical composition depends strongly on the fermentation substrate, microbial consortium, and fermentation duration, allowing the production of beverages with tailored chemical profiles and functional properties [17,64,65,66,67]. Organic acids are among the most abundant fermentation products and contribute to the characteristic flavour and biological activity of kombucha. Kombucha contains vitamins, amino acids, and proteins derived from both the tea infusion and microbial metabolism during fermentation. Vitamin C, together with several B-group vitamins (B1, B2, B6, and B12), have been identified among its micronutrient components [68,69,70]. In addition, a variety of amino acids, including leucine, lysine, valine, glutamine, and histidine, have been reported in fermented beverages and kombucha-derived products [71,72,73]. The levels of these compounds depend on fermentation conditions, substrate composition, and the microbial community involved, resulting in considerable variability among kombucha preparations. Additionally, the tea-derived polyphenols are considered key contributors to the biological activity of kombucha. The main groups include phenolic acids, flavonoids, and lignans. During fermentation, microbial enzymes can increase the release and bioavailability of phenolic compounds, thereby enhancing the antioxidant capacity of the beverage. However, excessive fermentation may result in the accumulation of organic acids and deterioration of beverage quality [74,75,76,77,78,79,80,81,82,83]. Kombucha also contains essential minerals, primarily derived from tea leaves and sugar. Commonly reported elements include potassium, magnesium, manganese, iron, zinc, copper, chromium, selenium, cobalt, and nickel [65].

The chemical composition of kombucha is particularly relevant to biomaterial applications, as many of its bioactive constituents may be transferred into the final biomaterial. This is relevant when kombucha liquid is used not only as a solvent or plasticising medium but also as a reactive fermentation-derived medium in which polymers are dissolved, dispersed, or cast. In this manner, compounds such as organic acids, polyphenols, vitamins, minerals, and microbial metabolites can become incorporated into the developing polymer matrix, potentially influencing its structural and functional properties [20,84,85]. Similarly, materials produced directly from the raw kombucha pellicle do not consist solely of bacterial cellulose but may also contain residual fermentation-derived compounds retained within the cellulose network. The BC pellicle develops at the air–liquid interface during kombucha fermentation as a highly porous biofilm matrix. This cellulose network serves as a structural scaffold in which microbial cells are embedded during biosynthesis, physically trapping bacteria, yeast, and fermentation-derived metabolites within the developing pellicle. Because of this, the final biomaterial may include cellulose fibrils as well as residual compounds from the fermentation medium, such as proteins, organic acids, polyphenols, and other low-molecular-weight metabolites. As a result, the pellicle can be considered a composite bio-based matrix that incorporates bioactive components during production, potentially enhancing the functional characteristics of products obtained from kombucha before purification treatments [19,86,87]. If retained after processing, these constituents may contribute to the biological activity of the resulting biomaterial, including antioxidant and antimicrobial effects, and therefore may play an important role in the performance of kombucha-based packaging systems.

The composition of bacterial cellulose (BC) obtained from kombucha is influenced not only by fermentation conditions but also by downstream processing. In particular, purification procedures determine the extent to which fermentation-derived compounds are retained within the cellulose matrix. Conventional alkaline treatments, commonly based on NaOH solutions, effectively remove microbial cells, proteins, pigments, and other non-cellulosic components, yielding a highly purified cellulose material with a reduced content of residual bioactive compounds [11,19,88,89]. As a result, the physicochemical and functional properties of kombucha-derived biomaterials may vary depending on the purification strategy applied after fermentation. The functionalities summarised in Table 1 are based on literature reports describing the activity of these compounds in biopolymer- and food-packaging systems. Although many bioactive constituents have been identified in kombucha and its pellicle, studies investigating their direct incorporation into packaging systems remain scarce. Current research is focused predominantly on bacterial cellulose isolated from the pellicle or on kombucha fermentation products used in combination with additional bioactive agents to improve material performance.

### 3.2. Antimicrobial and Antioxidant Properties Relevant to Packaging

The antimicrobial and antioxidant activities of kombucha depend on several interacting factors, including the fermentation substrate, microbial consortium, fermentation time, pH, organic acid concentration and transformation of tea-derived polyphenols. Organic acids, particularly acetic acid, are considered major contributors to antimicrobial activity; however, the greater inhibition exhibited by some kombucha preparations against selected microorganisms compared with equivalent acetic acid solutions indicates that polyphenols and other fermentation-derived metabolites may also contribute to this effect [1,116,117]. Antioxidant activity may increase during fermentation, owing to microbial transformation and release of phenolic compounds, but its development is not uniform and depends strongly on the substrate, starter culture and fermentation conditions. Prolonged fermentation may also result in the degradation of selected bioactive compounds [118,119,120]. For example, Greenwalt, Ledford and Steinkraus [116] observed antimicrobial activity for kombucha containing 33 g/L of total acids, including 7 g/L of acetic acid, supporting the importance of acidification in microbial inhibition. Chu and Chen [118] observed approximately 1.7-fold and 1.4-fold increases in DPPH- and ABTS-radical-scavenging activity, respectively, during fermentation; however, the magnitude of these changes depended on the tea substrate and fermentation time.

Nevertheless, antimicrobial or antioxidant activity demonstrated in the fermented beverage cannot be directly attributed to the raw pellicle, purified bacterial cellulose or final packaging material. Purified bacterial cellulose does not generally exhibit intrinsic antimicrobial or antioxidant activity. In contrast, native or mildly washed kombucha pellicles may retain organic acids, polyphenols and other fermentation-derived compounds and may therefore exhibit measurable biological activity [20,85]. Alkaline purification, repeated washing, drying and incorporation into a polymer matrix can substantially reduce or modify the concentration, stability and availability of these compounds [11,19]. Consequently, direct evaluation of biological activity and compound retention in the final packaging material, together with migration testing under relevant contact conditions, is necessary because these outcomes cannot be inferred from the properties of the kombucha beverage.

### 3.3. Packaging-Relevant Water, Thermal and Migration Properties

The hydrophilic character of bacterial cellulose is one of the principal limitations of its use in food packaging. The abundance of surface hydroxyl groups promotes moisture sorption, swelling and water uptake, which may increase water-vapour permeability and reduce dimensional and mechanical stability. Native kombucha-derived films produced using different plant infusions showed morphology-dependent differences in swelling and water-vapour transmission, with more porous films generally exhibiting poorer resistance to water [20]. Evidence obtained for broader bacterial-cellulose systems also demonstrates the importance of environmental humidity. In bacterial cellulose–chitosan–poly(vinyl alcohol) films, increasing water activity increased water-vapour permeability from 1.86 × 10^−12^ to 1.17 × 10^−11^ g m^−1^ s^−1^ Pa^−1^ and elongation from 3.25 to 36.55%, while reducing tensile strength and Young’s modulus [13]. Although this formulation was not produced specifically from kombucha-derived cellulose, it demonstrates why conditioning humidity and test conditions must be reported when KBC materials are compared.

Surface modification and crosslinking can improve water resistance, but the resulting effects are formulation-specific. Enzyme etching followed by cardanol–silane coating increased the water contact angle of KBC to 108.3 ± 0.4° and improved its resistance to water and water vapour [121]. Carbamate crosslinking of purified kombucha-derived BC reduced water retention and increased the onset temperature of thermal decomposition by 38–100 °C, depending on the crosslinking agent, whereas citrate treatment reduced tensile strength [10]. Similarly, the incorporation of 5 wt% oligo(lactic acid)-grafted KBC into PLLA and PHBV reduced oxygen permeability by approximately 23 and 45%, respectively, but increased the water-vapour permeability of PLLA by approximately 12% [122]. These results demonstrate that improvements in hydrophobicity, oxygen barrier or thermal stability do not necessarily translate into simultaneous improvement of all packaging properties.

Migration remains one of the least investigated aspects of KBC-based packaging. Native or mildly washed pellicles may release organic acids, polyphenols, microbial metabolites and residual medium components, whereas purified or chemically modified materials may contain residual purification agents, unreacted crosslinkers, coatings, active substances or non-intentionally added substances. Therefore, overall and specific migration should be tested using food simulants selected according to the intended food type, contact time and temperature. Evidence from general bacterial nanocellulose systems confirms that migration can be strongly dependent on use conditions. For example, ZnO-functionalised bacterial nanocellulose showed low zinc migration into food simulants and chicken skin at 4 °C, but the applicable migration limit was exceeded during contact with chicken skin at 10 and 22 °C [123]. At present, the available KBC packaging studies do not provide sufficient migration data to establish general food-contact compliance.

## 4. Food Packaging Applications

Kombucha-derived components can be incorporated into food-packaging systems through several distinct routes. Fermented kombucha liquid may be used as an acidic casting medium for dissolving film-forming polymers, particularly chitosan, whereas the cellulose pellicle may be applied as a native film, a purified bacterial cellulose matrix, or a filler or reinforcing phase in polymer composites [90,124,125]. These material forms are not interchangeable because their composition, purity, biological activity and performance depend strongly on fermentation conditions, purification and subsequent processing. Table 2 summarises the composition, processing and properties of kombucha-derived packaging materials, while Table 3 presents studies performed directly on food products.

Fermented kombucha liquid contains organic acids, polyphenols and other fermentation-derived compounds and can replace conventional acidic solvents in selected film-forming systems. For example, filtered kombucha liquid has been used to dissolve chitosan and produce films or coatings with formulation-dependent mechanical, barrier and antimicrobial properties [90,131]. However, the composition of the liquid varies with the fermentation substrate, microbial consortium and fermentation time. Its antioxidant or antimicrobial activity therefore cannot be assumed to remain unchanged after incorporation into a film, and the retention, stability and migration of active compounds should be evaluated directly in the final packaging material.

Purified kombucha-derived bacterial cellulose provides an interconnected nanofibrillar network with generally high crystallinity and desirable film-forming properties [11,125]. It may be used as a standalone structural matrix or incorporated into polymers such as PLA, PHBV, agar, alginate and CMC. Nevertheless, its effect is strongly dependent on filler concentration, dispersion, interfacial compatibility and processing. Some studies reported increased tensile strength or stiffness after KBC incorporation or modification, whereas others observed reduced tensile strength, elongation or water-vapour resistance [122,132,133]. For example, modified KBC improved the oxygen barrier of PLLA and PHBV composites but simultaneously increased water-vapour permeability [122].

Native or mildly washed pellicles may retain polyphenols and other fermentation-derived compounds and can therefore exhibit antioxidant or antimicrobial activity [20,85]. In contrast, alkaline purification improves cellulose purity and reproducibility but may remove compounds contributing to the activity of the untreated pellicle. Verification of active properties after purification, drying and incorporation into the final polymer matrix is therefore necessary. Direct food studies remain relatively limited and include applications involving minced beef, yoghurt, grapes, plums, tomatoes, peppers and other fresh products [124,127,128,130,131,139]. Although several studies reported delayed quality deterioration, microbial inhibition or successful freshness monitoring, direct comparison remains difficult because of differences in storage temperature, duration, controls and quality criteria. Further studies should therefore include standardised storage conditions, conventional-packaging controls, migration and food-contact safety assessments, and direct measurements of the stability and release of active compounds. Although outside the direct scope of food packaging, kombucha liquid and residual SCOBY biomass have also been investigated as fermentation starters, hydrocolloids or ingredients in dairy beverages, bread, smoothies, jams, confectionery, tempeh and fermented pollen products [119,140,141,142,143]. These routes may contribute to the integrated valorisation of co-products generated during KBC production but are not discussed further here.

## 5. Comparative Assessment of Production and Modification Strategies for Kombucha-Derived Bacterial Cellulose

Approaches to producing and modifying kombucha-derived bacterial cellulose (KBC) differ considerably in terms of packaging performance, scalability, and suitability for food-contact applications. Native or purified KBC films provide a continuous nanofibrillar cellulose network and can exhibit favourable mechanical and oxygen-barrier properties under dry conditions. Their performance, however, depends strongly on the microbial consortium, fermentation medium, purification procedure, drying method, film thickness, and conditioning humidity [4,20,85]. In particular, its hydrophilic surface promotes water uptake and swelling, which can reduce mechanical integrity and barrier performance at elevated relative humidity [12,13,144]. Kombucha beverage has also been used as an acidic casting medium for chitosan films and coatings, where it may contribute organic acids and polyphenols; nevertheless, these solvent-cast systems should be distinguished from films in which KBC forms the structural matrix [90,131]. The broad range of tensile strength values reported for KBC materials (30.3–128.9 MPa) cannot be attributed solely to inherent variability in bacterial cellulose [4]. Differences may result from pellicle purification, fibril organisation, residual compounds, drying procedure, film thickness, conditioning humidity, specimen geometry and the mechanical-test method used.

Modification strategies can be introduced during biosynthesis (in situ) or after purification (ex situ). In situ incorporation may reduce the number of downstream steps and promote integration of selected compounds within the cellulose network, but it can also alter microbial growth, cellulose yield, crystallinity, and fibril organisation. Ex situ blending, coating, esterification, and crosslinking offer greater control over the final formulation and have improved water resistance, flexibility, or active functionality in selected KBC systems [10,121,145]. These improvements are formulation-specific and may increase process complexity, solvent and chemical use, cost, migration concerns, or the time required for regulatory assessment. Consequently, no current approach simultaneously optimises moisture resistance, mechanical performance, biodegradability, safety, and industrial scalability. The evidence base and principal trade-offs are summarised in Table 4.

## 6. Comparison with Conventional Packaging Polymers

Studies have shown that kombucha-derived bacterial cellulose can achieve tensile strength comparable to or greater than that of commonly used packaging polymers. Native KBC films produced using different herbal infusions exhibited tensile strengths ranging from 30.3 to 128.9 MPa, whereas approximately 14 MPa was reported for a representative polyethylene film, and PP and PLA packaging films showed values of 25–31 MPa and 50–58 MPa, respectively [4,165,166]. However, such comparisons are influenced by differences in polymer grade, processing, film orientation and thickness, conditioning humidity, and tensile-test methodology among studies. Regarding barrier performance, hydrophobic PE and PP generally provide more reliable resistance to water vapour, whereas the hydrophilic structure of KBC promotes moisture sorption and swelling and may impair its mechanical and barrier performance at elevated relative humidity. On the other hand, the dense nanofibrillar structure of dried bacterial cellulose can provide desirable oxygen-barrier performance under dry conditions, while PLA generally exhibits intermediate gas- and water-vapour-barrier properties that depend strongly on crystallinity, processing and environmental humidity [20,167,168]. The materials also differ considerably in their end-of-life behaviour: unmodified PE and PP are environmentally persistent and are not considered biodegradable under conventional environmental or composting conditions, whereas PLA biodegradation generally requires controlled industrial-composting conditions. For example, PLA bottles reached 77.8–84.2% mineralisation after 58 days at 58 °C, while unmodified kombucha-derived BC exhibited a mass loss of 45.2% after 21 days of soil burial [108,169,170]. However, mineralisation and soil-burial mass loss represent different degradation endpoints; therefore, these values illustrate general differences in end-of-life behaviour rather than directly comparable degradation rates.

## 7. Research Gaps, Limitations and Future Directions of Kombucha-Based Packaging

Although KBC is a promising renewable packaging material, translation from laboratory films to food-contact products remains limited by process variability, moisture sensitivity, incomplete safety assessment, and a lack of pilot-scale and real-food validation. Future work should prioritise standardised material definitions and test protocols, scalable production, food-contact compliance, and comparisons performed under realistic storage conditions.

### 7.1. Scalability and Standardisation

Most KBC studies use small, static cultures, whereas industrial production requires control of oxygen transfer, vessel geometry, inoculum preparation, fermentation time, and downstream handling at a substantially larger scale [171]. Published results also differ in consortium composition, substrate formulation, purification, drying, and conditioning, which limits direct comparison of cellulose yield and material performance [172,173]. The absence of broadly accepted specifications for KBC—including dry-mass yield, residual microbial and non-cellulosic content, degree of purification, thickness, moisture content, and test humidity—remains a major obstacle to batch-to-batch reproducibility. Commercial and prototype initiatives have been reported; however, peer-reviewed evidence for continuous, large-volume production of food-contact KBC remains scarce. A reliable assessment of scale-up therefore requires studies to report mass and energy balances, productivity normalised to reactor volume and exposed surface area, water and chemical demand during purification, drying-energy requirements, and the effects of process intensification on fibril structure and film performance. Where beverage and KBC are intended as co-products, process optimisation must also consider whether additives used to enhance cellulose production or functionality adversely affect beverage quality [174].

### 7.2. Techno-Economic Feasibility

The most directly relevant techno-economic study modelled a kombucha-based cellulose facility with an annual capacity of 60 t. The estimated total capital investment was approximately USD 13.72 million, annual operating costs were approximately USD 3.8 million, and the production cost of dry cellulose was approximately USD 63.8 kg^−1^. The simulated payback period, return on investment, and internal rate of return were 4.23 years, 23.64%, and 16.48%, respectively [175]. These values describe a modelled process rather than a validated commercial plant and were particularly sensitive to labour, facility-dependent expenditure, fermentation efficiency, and downstream processing. Dourado, et al. [176] reported a lower modelled cost of approximately USD 14.8 kg^−1^ for a 504 t year^−1^ bacterial nanocellulose facility using beet molasses. However, that analysis concerned industrial bacterial nanocellulose rather than kombucha-derived cellulose and used a different scale, feedstock, and process configuration. The two estimates therefore illustrate the influence of production assumptions but cannot be interpreted as directly comparable estimates of KBC production costs. Similarly, favourable cost estimates reported for other nanocellulose processes cannot be regarded as representative of KBC production costs. Robust assessment requires pilot-scale data and sensitivity analyses covering substrate price, labour, utilities, fermentation duration, purification chemicals, water use, drying, product losses, and automation. Environmental performance also cannot be inferred from biodegradability alone. Life-cycle studies of bacterial cellulose show that the culture medium, electricity demand, water use, purification, and particularly drying and downstream processing can substantially influence environmental impacts [177]. Moreover, degradation rates depend on material composition and test conditions; a value obtained for a particular BC composite in soil cannot be generalised to all KBC films. Robust KBC-specific life-cycle assessments require comparison with conventional and bio-based alternatives on the basis of equivalent packaging functions and shelf-life performance, together with realistic end-of-life scenarios, rather than reliance solely on laboratory biodegradation data.

### 7.3. Food-Contact Safety and Regulatory Requirements

In the European Union, all KBC-based food-contact materials must satisfy the general safety, inertness and traceability requirements of Regulation (EC) No 1935/2004 and be produced according to the good-manufacturing-practice requirements of Regulation (EC) No 2023/2006 [178]. When KBC is incorporated into a plastic matrix or forms part of a plastic multilayer structure, Regulation (EU) No 10/2011, as subsequently amended, may also apply [179]. For materials covered by this Regulation, the overall migration limit is 10 mg dm^−2^ of food-contact surface, while specific migration limits apply to individual authorised substances. However, KBC systems designed to release active compounds or to monitor food quality may additionally fall within Regulation (EC) No 450/2009, concerning active and intelligent materials [180]. In the United States, there is no single universal migration limit applicable to every food-contact material. The regulatory status of each component depends on its identity, intended use and expected dietary exposure. Depending on the material and existing authorisations, the appropriate pathway may include an effective Food Contact Notification, a food-additive regulation, a Threshold of Regulation exemption or another legally applicable status. The FDA evaluates the identity of migrating substances, predicted migration and cumulative dietary exposure together with toxicological evidence [181]. Most published KBC studies report mechanical, barrier or antimicrobial performance without performing overall migration, specific migration or comprehensive identification of non-intentionally added substances. Consequently, currently available results do not demonstrate that KBC packaging materials as a group comply with either EU or US food-contact requirements. Future studies should evaluate migration under the intended time–temperature conditions and include residual cells, fermentation metabolites, purification chemicals, crosslinkers, coatings and active compounds in the safety assessment.

### 7.4. Performance Under Realistic Storage and Distribution Conditions

Most reported KBC films have been evaluated at laboratory scale under controlled temperature and relative humidity. These conditions do not fully reproduce variations encountered during food filling, refrigerated or ambient storage, transport, and retail. Because cellulose is hydrophilic, increasing relative humidity can increase water-vapour transmission and reduce tensile performance, oxygen-barrier efficiency, and dimensional stability [12,13,23]. The performance of KBC in contact with high-moisture, acidic, fatty, or enzymatically active foods also remains insufficiently characterised. Polymer reinforcement, coatings, and chemical modification may improve moisture resistance, but these benefits must be assessed together with possible reductions in biodegradability, recyclability, or food-contact acceptability. Future studies would benefit from the use of matched unmodified controls, standardised test methods, relevant humidity gradients, and real foods. Particular research attention is warranted for applications in which KBC provides measurable improvements in shelf life or product quality, rather than merely desirable properties in isolated-film tests.

### 7.5. Consumer Acceptance and Market Launch

Direct evidence concerning consumer acceptance and willingness to pay for KBC food packaging is currently lacking. Studies of other bio-based and biodegradable packages indicate that environmental benefits alone do not determine purchasing decisions; price, perceived safety, functionality, disposal instructions, and familiarity with the material are also important [182,183,184]. These findings provide only indirect guidance for KBC. Consumer acceptance of KBC-based packaging remains insufficiently characterised and cannot be inferred from studies of kombucha beverages or edible pellicle products. Dedicated studies are needed to determine how microbial origin, material appearance and texture, labelling, food-contact safety information and substantiated end-of-life claims affect consumer perception.

## 8. Biodegradability and End-of-Life Considerations of KBC

Although bacterial cellulose is susceptible to enzymatic degradation by cellulolytic microorganisms, its biodegradability cannot be extrapolated to every KBC-based film or composite. The degradation rate depends on material thickness, crystallinity, porosity, moisture availability, microbial activity and test environment. Blending, crosslinking, hydrophobic coating and incorporation of antimicrobial substances may additionally restrict water penetration or microbial colonisation and thereby reduce the degradation rate. Conversely, the incorporation of KBC into a biodegradable polymer may increase water uptake and facilitate degradation of the composite matrix.

Direct evidence for kombucha-derived materials remains limited and shows that the result depends strongly on formulation and test conditions. In a 21-day soil-burial experiment, unmodified kombucha-derived BC exhibited a mass loss of 45.2%, whereas a crosslinked composite containing glycine, calcium chloride and cinnamaldehyde showed a lower mass loss of 32.4% [108]. The slower degradation of the modified material was attributed to its crosslinked structure and antimicrobial components. Chong, Cheung, Jiang and Ngai [121] reported that enzyme-etched KBC coated with cardanol-based silane retained biodegradability and completely degraded within five months under the conditions used in their study. In another approach, the incorporation of 5 wt% oligo(lactic acid)-grafted KBC increased the biodegradation rate of PLLA- and PHBV-based composites evaluated using a procedure based on EN 13432 and OECD 301B [122]. Taken together, these findings indicate that degradation behaviour varies among formulations and test conditions, making it difficult to assign a universal degradation time to KBC packaging. Soil-burial mass loss, visual disintegration and carbon dioxide evolution assess different aspects of degradation and are therefore not directly comparable. In particular, partial mass loss in soil alone is insufficient to demonstrate industrial compostability. Standardised evaluation therefore remains necessary, including mineralisation testing according to ISO 14855-1 and assessment of industrial compostability against the relevant EN 13432 criteria, together with reporting of disintegration, ecotoxicity and the chemical characterisation of remaining residues [185,186]. The environmental advantage of KBC should also be evaluated using life-cycle assessment because fermentation media, purification, water consumption and drying may contribute substantially to the overall environmental impact of bacterial-cellulose production [177,187].

## 9. Conclusions

Kombucha fermentation provides several distinct routes for the development of food-packaging materials. The fermented beverage can serve as an acidic casting medium containing fermentation-derived compounds, whereas the raw pellicle represents a hydrated cellulose-based biofilm containing microbial cells and residual components of the cultivation medium. Purified kombucha-derived bacterial cellulose (KBC), in contrast, is a more chemically defined structural material. Purified KBC offers a continuous nanofibrillar network and can provide favourable mechanical and oxygen-barrier properties under dry conditions. Its pronounced hydrophilicity, however, promotes moisture sorption and swelling and may impair mechanical integrity and barrier performance at elevated relative humidity. Blending, coating, grafting and crosslinking can improve selected properties, but the effects are formulation-specific and may involve trade-offs. An improvement in hydrophobicity, oxygen-barrier performance or thermal stability does not necessarily produce a simultaneous improvement in water-vapour resistance, strength, biodegradability or food-contact safety. Consequently, KBC cannot be assigned a single set of representative material properties without considering the microbial consortium, cultivation conditions, purification procedure, drying method, film thickness and conditioning conditions. Evidence supporting the antioxidant and antimicrobial potential of kombucha-derived packaging is promising but remains inconsistent. Biological activity measured in the beverage or raw pellicle cannot be automatically attributed to purified KBC or to the final packaging material. The retention, stability, release and effectiveness of fermentation-derived compounds must therefore be evaluated directly in each formulation. Moreover, relatively few studies have examined real food products, migration, residual microorganisms, purification chemicals, non-intentionally added substances or performance during realistic storage. Similarly, degradation observed in soil-burial or other laboratory tests alone is insufficient to demonstrate industrial compostability, which requires evaluation under recognised standards. Key priorities for future research include standardised material terminology, transparent reporting of cultivation and purification conditions, controlled-humidity property testing, migration studies and application-specific trials using food simulants and real foods. Meaningful pilot-scale evaluation requires integrated consideration of oxygen transfer, reactor design, water and chemical consumption, purification efficiency, production costs and life-cycle assessment. Regulatory compliance and consumer acceptance are also integral to material development and cannot be deferred until after formulation optimisation. On the basis of current evidence, KBC appears particularly promising as a reinforcing phase, coating component, structural matrix or carrier of active compounds in carefully designed packaging systems. However, further standardisation, safety assessment and scale-up evidence are required before it can be regarded as a broadly applicable replacement for conventional food-packaging plastics.

## Figures and Tables

**Table 1:** Major Constituents of Kombucha and Their Potential Relevance to Active Food-Packaging Materials.

Fermentation-Derived Compound Group	Compounds Reported in Kombucha Beverage or Raw Pellicle	Analysed Sample	Representative Quantitative Data	Potential Relevance to Packaging Materials	Ref.
Organic Acids	Acetic, gluconic, glucuronic, citric, L-lactic, malic, tartaric, malonic, oxalic, succinic, pyruvic Quinate, propionate, chloride	Liquid kombucha beverages prepared from green or black tea	Acetic acid: up to 9.51 g/L by day 15 Lactic acid: ~0.54 g/L by day 3 Glucuronic acid: up to 2.33 g/L by day 12	Selected short-chain organic acids may act as natural plasticisers that disrupt native hydrogen bonding, which increases film flexibility and elongation at break Residual acids establish a low-pH microenvironment within the hydrogel, which exerts direct antimicrobial activity against food spoilage pathogens	[3,7,57,76,87,90,91,92,93,94,95,96]
Vitamins	Vitamin C (ascorbic acid), thiamine (B1), riboflavin (B2), B6, cobalamin (B12)	Liquid yellow-marigold flower kombucha	Vitamin C: up to 754.8 mg/L	Ascorbic acid functions as a potent endogenous oxygen scavenger within the matrix, which helps to protect oxygen-sensitive packaged foods from oxidative degradation Specific vitamins, such as riboflavin, can contribute to the absorption of ultraviolet radiation—this shielding effect protects light-sensitive foods	[18,20,87,97,98,99,100,101]
Amino Acids & Proteins	L-theanine, leucine, lysine, valine, glutamine, histidine, proline, arginine	Dried raw pellicle (lysine); liquid kombucha beverage (soluble protein)	Lysine: up to 53.1 mg/g DW Proteins: ~1.7–5.0 mg/L	Proteins and amino acids associated with the raw pellicle may interact with cellulose through hydrogen bonding and influence mechanical properties. However, their retention after washing and their contribution to KBC film performance remain unverified	[102,103,104,105]
Tea-Derived Polyphenols	Phenolic acids (gallic, p-Coumaric), flavonoids (quercetin, vitexin), catechins (epigallocatechin)	Liquid black-tea kombucha beverage	Total Polyphenols (TPC): 0.42 mg/mL GAE Gallic acid: up to 30.23 μg/mL	When retained in the final material, tea-derived polyphenols may provide radical-scavenging activity and contribute to a reduction in UV transmission. Their retention and activity must be verified after washing, drying and film formation	[20,106,107]
Carbon Dioxide (CO_2_)	Carbon dioxide (CO_2_)	Liquid kombucha beverage	CO_2_: up to 152 mg/L	CO_2_ bubbles may contribute to the development of pores within the forming pellicle; however, their direct effect on the gas-barrier properties of the final material has not been sufficiently demonstrated	[7,57,108,109,110,111]
Minerals	Zinc, manganese, copper, iron, potassium, magnesium, cobalt, nickel, chromium, selenium	Liquid kombucha beverages prepared from different tea types	Zinc: up to 2.08 mg/L Manganese: up to 1.40 mg/L	Multivalent ions can alter cellulose-network interactions when intentionally introduced as crosslinkers. However, this effect has not been demonstrated for the trace concentrations measured in kombucha beverages, nor has their retention in purified KBC been established	[16,65,108,112,113,114,115]

**Table 2:** Composition, processing and reported performance of kombucha-derived food-packaging materials.

Matrix/Format	Kombucha-Derived Fraction	Treatment and Loading	Mechanical Performance	Barrier/Thermal Performance	Active, Intelligent or Other Function	Reference
Chitosan film	Fermented black-tea kombucha liquid	No clarification of cell removal or pretreatment; solution casting; 50 °C, 24 h	NR	WVP: 256.7 → 132.1 g · mm · cm^−2^ · h^−1^ · kPa^−1^; improved UV protection	DPPH scavenging up to 59%; antimicrobial effect demonstrated in a food test	[124]
Alginate–anthocyanin film; Pickering emulsion	KBC nanocrystals from black-tea pellicle	Grinding; 50% H_2_SO_4_ hydrolysis; dialysis; freeze-drying. CBPE: 0.1–0.4% in the film formulation	TS: 12.41 → 32.76 MPa	Lower transmittance at 200–800 nm	DPPH and ABTS scavenging ≈ 33%; anthocyanin-based pH response	[126]
CMC–pomegranate anthocyanin film/coating	Nanofibrillated KBC from black-tea pellicle	1 M NaOH, 90 °C, twice; homogenisation. KBC: 1–15 wt% relative to CMC	TS: 1.28 → 18.51 MPa	UV blocking: 28.2% → 100%	Antimicrobial activity; visible colour response from pH 2 to 12	[127]
Standalone KBC film; glycerol- or chitosan-treated variants	KBC from black-tea waste	2 M NaOH, 90 °C, 2 h; 2 M NaClO, 2 h; pressure dewatering and hot-air drying	Approx. thickness 0.14 mm; maximum load ≈ 140 N; TS ≈ 100 MPa	NR	Food-contact film	[128]
Standalone dried SCOBY film	Washed black-tea pellicle	Water washing and sun-drying; optional olive-oil/beeswax surface treatment	NR	Leakage resistance assessed qualitatively	Activity reported against *Bacillus*, *Enterobacter*, *Staphylococcus* and *Pseudomonas*	[129]
BNC film with or without chitosan	Purified black-tea BNC pellicle	0.5 N NaOH; pressing; drying at 60 °C; immersion in 1% chitosan for 48 h	TS: 23.56 → 33.13 MPa; tear strength: 57 → 63 mN	NR	Antibacterial activity against *E. coli* and *A. viridans*	[130]
Chitosan film and edible coating	Cell-free lemon-balm kombucha liquid	Centrifugation and membrane filtration; 1% (w/v) chitosan; casting or dipping	TS 11.08 MPa; EAB 53.45%	WVTR 131.84 g·m^−2^·d^−1^	TPC 381.67 µg GAE·mL^−1^ in solution; antimicrobial activity in vitro	[131]
Chitosan film	Cell-free kombucha liquids from black, green and white tea or coffee	Centrifugation and membrane filtration; 1% (w/v) chitosan; room-temperature casting	CWF: TS 4.89 MPa, EAB 24.46%; CBF: EAB 74.5%	OTR: 1.25–5.40 cm^3^·m^−2^·d^−1^; WVTR: 140.8–153.2 g·m^−2^·d^−1^	Formulation-dependent antibacterial activity	[90]
Native standalone KBC film	Water-washed pellicles from black/green tea, rosehip, coffee and licorice infusions	Washed to pH 7.0 ± 0.2; dried at 105 °C for 2 h; no alkaline purification	EAB: 10.90–25.24%	WVTR generally > 50 and up to ≈85 g·m^−2^·d^−1^	Green-tea film: DPPH 74.22 ± 2.05%, ABTS 81.59 ± 2.39%; rosehip film: ABTS 83.37 ± 0.63%; antimicrobial activity	[20]
Agar–alginate film	Intact, enzymatically hydrolysed or VFD-treated KBC; 2.5 wt%	1 M NaOH, acetic-acid wash, cellulase hydrolysis; optional VFD at 6000 rpm; casting	Control 9.98 MPa; intact KBC 7.69 MPa; hydrolysate 11.11 MPa; VFD hydrolysate 18.18 MPa	NR	Biodegraded within 5 d; VFD improved strength without compromising biodegradability	[132]
PLA–maleinised linseed oil film	KBC from pristine (CK) or spent-coffee (SCK) infusion; 3 or 5 wt%	Autoclaving, homogenisation, ultrasonication, drying and milling; twin-screw extrusion	PLA–MLO: E 1308.4 MPa, TS 12.9 MPa; 5% SCK: E 1639.2 MPa, TS 31.2 MPa; 5% CK: E 916.7 MPa, TS 7.5 MPa	WVTR: 82.3 (PLA–MLO) → 110.6–118.1 g·m^−2^·d^−1^ in 5% KBC films	DPPH: 37.08 ± 4.77% (5% CK) and 12.85 ± 0.68% (5% SCK); clear strength–barrier trade-off	[133]
Chemically crosslinked standalone KBC film	Purified black-tea KBC pellicle	NaOH/NaOCl purification; citrate or diisocyanate-mediated carbamate crosslinking	Untreated TS 25.3 ± 1.8 MPa; citrate-treated 7.5 MPa; toluene-linked 51.3 ± 5.4 MPa	Carbamate treatment reduced water retention; exact values formulation-dependent	Decomposition onset increased by 38–100 °C after carbamate crosslinking	[10]
PLLA or PHBV nanocomposite film	OLLA-grafted KBC from SCOBY; 5 wt%	NaOH purification; freeze-drying; oligo(lactic acid) grafting; extrusion and hot pressing	Young’s modulus: PLLA +≈12%; PHBV −≈14%; TS changes were not significant	OP: PLLA 18.3 → 14.1; PHBV 5.0 → 2.8 cm^3^·mm·m^−2^·d^−1^·atm^−1^. WVP: PLLA 1.06 → 1.19; PHBV 0.13 → 0.17 g·mm·m^−2^·d^−1^·kPa^−1^	Improved oxygen barrier but slightly higher water-vapour permeability	[122]
Cellulose substrate biocoated with growing kombucha biofilm	Liquid black-tea SCOBY culture	Cellulose pad incubated in kombucha culture for 14 d	TS reached a maximum on day 10; EAB decreased from ≈2% to ≈0.6%	NR	Antimicrobial activity against *E. coli* and *S. aureus*	[134]
Modified standalone KBC composite	Alkali-purified KBC pellicle	0.5% BAC50, 1% glycine, 1% CaCl_2_ and 0.5% cinnamaldehyde; air-drying	TS: 125 ± 5 → 275 ± 5 MPa; tensile modulus: 4.2 ± 0.2 → 8.9 ± 0.3 MPa	OTR: 1789 ± 70 → 433 ± 25 cc·m^−2^·d^−1^ (≈75.8% decrease, calculated); WVTR 0.283 g·m^−2^·d^−1^	Antimicrobial activity against *E. coli* and *S. aureus*; non-standard/manual test setups limit comparability	[108]
Native standalone KBC film	Pellicles from six herbal infusions	Heat inactivation, drying, rehydration and overnight casting	TS: 30.3 MPa (oregano) to 128.9 MPa (yerba mate)	NR	Yerba-mate film: ABTS radical inhibition 93 ± 4%	[4]
Chitosan-coated KBC film	Purified green-tea KBC	1 N NaOH; chitosan coating at 0.5, 1.0 or 1.5%; drying at 55 °C	NR	Improved UV–Vis light absorption; opacity up to 75.24%	Maximum reported scavenging: DPPH 57.71%, ABTS 24.57%; inhibition zones: *S. aureus* 6.55 mm, *E. coli* 8.25 mm	[135]
Plasticised mechanically recycled PLA film	KBC from pristine yerba mate (KMN) or yerba-mate waste (KMW); 1 or 3 wt%	Autoclaving, homogenisation, drying and milling; 15 wt% ATBC; solvent casting	At 1 wt% KBC: E ≈ 750–850 MPa and TS ≈ 15–17 MPa; EAB up to 17% for KMN	KBC increased WVTR; KMW films had lower WVTR than KMN films	KMN films showed greater antioxidant release than KMW films; no activity in unloaded r3-PLA	[136]
Kombucha-powder/starch/glycerol film with parsley extract	Dried and milled black-tea SCOBY; 5 g per 100 mL casting liquid	Drying at 80 °C, milling, mixing at 60 °C and room-temperature casting	Starch increased TS to about 3 MPa; glycerol increased EAB up to about 70%	Parsley extract and starch improved the reported water-vapour barrier	Pure film: DPPH 40 ± 2%; parsley significantly enhanced antioxidant and antibacterial activity	[137]
PLA film	Purified and micronised black-tea KBC; 1, 3 or 5 wt%	NaOH/NaOCl purification, drying, milling and melt processing	Ultimate TS: 67 MPa (PLA) → 66, 62 and 57 MPa at 1, 3 and 5 wt% KBC	NR	KBC acted as a filler but did not reinforce tensile strength under the reported processing conditions	[138]

Abbreviations: ABTS, 2,2′-azino-bis(3-ethylbenzothiazoline-6-sulfonic acid); ATBC, acetyl tributyl citrate; BNC, bacterial nanocellulose; CBPE, cellulose-stabilised Pickering emulsion; CBF/CCF/CGF/CWF, chitosan films prepared with black-tea/coffee/green-tea/white-tea kombucha; DPPH, 2,2-diphenyl-1-picrylhydrazyl; E, tensile modulus; EAB, elongation at break; KBC, kombucha-derived bacterial cellulose; OP, oxygen permeability; OTR, oxygen transmission rate; TS, tensile strength; VFD, vortex fluidic device; WVP, water-vapour permeability; WVTR, water-vapour transmission rate; NR, not reported.

**Table 3:** Direct tests of kombucha-derived packaging materials on food products.

Material	Food Product	Storage	Main Results	Reference
Chitosan–kombucha liquid film	Fresh minced beef	Refrigerated storage; 6 d	Extended the reported acceptable storage period to 6 d and reduced *S. aureus* counts.	[124]
Alginate–anthocyanin–CBPE indicator film	Yoghurt	Freshness monitoring	Colour response tracked pH change. This supports intelligent-packaging use.	[139]
CMC–anthocyanin/KBC film or coating	Red Globe grapes; African Rose plums	Up to 25 d	Wrapping/coating delayed visible quality deterioration for up to 25 d.	[127]
Dried KBC films, including glycerol- and chitosan-treated variants	Tomatoes	Accelerated storage	Reported extension of tomato shelf life by 13–15 d.	[128]
Washed, sun-dried SCOBY films	Tomatoes, spinach and grapes	Incubation up to 8 d	No visible spoilage was reported through day 8.	[129]
BNC and BNC–1% chitosan films	Tomatoes	Up to 28 d	BNC-wrapped tomatoes remained fresh to day 20; BNC–chitosan samples remained fresh and firm through day 28 without visible microbial spoilage. Polythene and unwrapped controls deteriorated earlier.	[130]
Chitosan–lemon-balm kombucha coating	Red bell pepper	18 °C; 15 d	Coated peppers had 32% higher initial skin strength and improved elasticity through day 10; ascorbic acid was 139.0 vs 131.7 mg·100 g^−1^ FW.	[131]

Only direct food-product tests are included. In vitro antimicrobial or antioxidant assays, pH response in buffer, and material characterisation alone were not treated as evidence of food shelf-life extension.

**Table 4:** Comparative assessment of available production and modification approaches for KBC-based materials.

Approach/Method	Description	Advantages	Disadvantges/Challenges	Ref.
Native BC films	films produced directly from kombucha fermentation without additional compounds	high mechanical strength; high flexibility; biodegradable; sustainable	hydrophilic nature leads to high WVP; variability with thickness and porosity; variability of batch-to-batch production	[20]
Polymer composites/blends	KBC combined with polymers such as chitosan, PLA, starch	improved tensile strength, water and oxygen barrier; antimicrobial properties (dependent of used polymer)	possible reduced biodegradability; higher production cost regulatory concerns; processing complexity	[138,146,147,148]
Chemical/enzymatic modifications	surface modification of BC (e.g., esterification)	high-performance films; adjustable properties for specific applications	complex post-processing of BC; scaling challenges; potential cost increase; regulatory issues	[14,121]
Waste-derived/upcycled BC	usage of agro-industrial waste as source for BC production	reduced substrate cost; compliant with circular economy; sustainable	variability in microbial growth and BC quality; standardisation issues; need of process optimisation	[149,150,151]
Bioactive compounds/nanoparticles	implantation of antimicrobial agents (silver nanoparticles, essential oils, plant extracts) into BC	adds active packaging functionality (antimicrobial, antioxidant); reduced WVTR	higher production cost; regulatory concerns; may affect biodegradability	[24,152,153]
Freeze-dried/Aerogel BC films	producing porous, lightweight BC aerogels or foams through freeze-drying	low density; high surface area; potential for active packaging	expensive; energy-intensive; mechanical strength may decrease with time	[109,154,155,156]
Layer-by-layer (LbL) coatings	coating BC films with layers of polymers (e.g., chitosan, alginate)	possibility to modify water/oxygen barrier properties; improved mechanical stability	complex processing; scaling up may be challenging	[157,158,159]
Enzymatic crosslinking	usage of enzymes (e.g., transglutaminase) to crosslink BC or BC composites	enhanced mechanical and barrier properties; no chemical additives	cost of enzymes; optimisation required; potential impact on biodegradability	[160,161,162,163]
In Situ Hybridisation/Functional Growth	incorporation of additives during fermentation (bioactive agents, polymers, etc.)	possible KBC property improvement (antimicrobial, antioxidant); less post-processing requirement	chemical evaluation of used additives; possible effect on fermentation and yield	[164]

## Data Availability

No new data were created or analyzed in this study. Data sharing is not applicable to this article.
